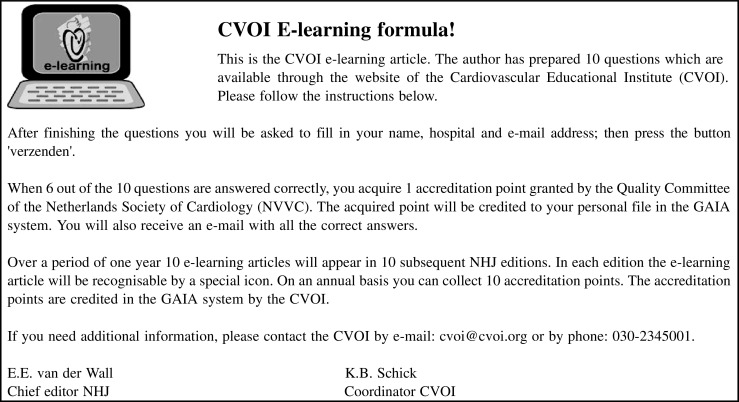# Negative predictive value of SPECT for the occurrence of MACE in a medium-sized clinic in the Netherlands

**DOI:** 10.1007/s12471-014-0524-1

**Published:** 2014-02-27

**Authors:** M. J. Bom, J. M. B. Manders, R. Uijlings, E. A. Badings, F. M. A. C. Martens

**Affiliations:** 1 Department of Cardiology, Deventer Hospital, N. Bolkesteinlaan 75, 7416 SE Deventer, the Netherlands; 2 Department of Nuclear Medicine, Deventer Hospital, N. Bolkesteinlaan 75, 7416 SE Deventer, the Netherlands

**Keywords:** Single photon emission computed tomography, SPECT, Prognosis, Major adverse cardiac events, MACE, CAD

## Abstract

**Background:**

Single-photon emission computed tomography (SPECT) is an important prognostic tool in evaluating coronary artery disease (CAD), with a high negative predictive value (NPV) for the occurrence of major adverse cardiac events (MACE). The prognostic value of SPECT is disputed in women, patients with atrial fibrillation (AF), diabetes, left bundle branch block (LBBB) and renal impairment.

**Methods:**

Seven hundred sixty-two patients without prior history of CAD who had SPECT without perfusion deficits were followed for 2 years for MACE. Predictive variables for the occurrence of MACE were reviewed by Cox proportional hazard regression, considering clinical information, resting-ECG data and SPECT data.

**Results:**

The NPV of SPECT for the occurrence of MACE within 2 years was 95.8 %. Multivariate Cox regression revealed male gender as the only significant predictor for the occurrence of MACE, besides a positive stress ECG at SPECT and a low LVEF. AF, LBBB, renal impairment and diabetes had no significant effect on the prognosis after normal SPECT.

**Conclusion:**

SPECT with normal perfusion images has great NPV in a medium-sized clinic in the Netherlands, even in patients with LBBB, AF, diabetes and renal impairment. MACE-free survival, however, was negatively influenced by male gender; we therefore propose more caution in men.

## Introduction

Ischaemic heart disease is the second cause of death in the Netherlands, and has a high morbidity and mortality rate [1, 2]. The main cause of myocardial ischaemia is coronary artery disease (CAD) [3]. Single-photon emission computed tomography (SPECT) is an important imaging modality in the analysis of functional ischaemia in patients with suspected CAD [3]. SPECT is reported to have great prognostic value for the occurrence of cardiac events [4–9], with an annual myocardial infarction (MI) rate after negative SPECT of 0.8 % [7]. An important limitation in the use of SPECT is the reduced sensitivity of balanced ischaemia in three-vessel CAD [10].

There have been doubts about the prognostic value of SPECT in certain subgroups. Studies have shown that women [7], patients with renal impairment (eGFR<60) [11–13] and patients with atrial fibrillation (AF) [14, 15] have a higher event rate after normal SPECT. Furthermore, the prognostic use of SPECT in diabetic patients and patients with left bundle branch block (LBBB) has also been disputed.

Recent studies have shown similar event rates after normal SPECT in diabetics compared with non-diabetics [16], although balanced triple-vessel disease is more common in diabetics [17] and thus may cause underestimation of functional ischaemia on SPECT.

Patients with LBBB have intrinsically abnormal conduction and are reported to have a related pattern of perfusion abnormalities. A recent study, however, has shown that prognosis of a normal SPECT was not altered by LBBB [18].

Population definition and pre-test likelihood are of great influence on reported prognosis and thus it is crucial to have representable data about our specific population.

## Aim

The aim of this study was therefore to evaluate the negative predictive value (NPV) of combined exercise and pharmacological stress SPECT with attenuation correction (AC) in patients without a prior history of CAD in a Dutch hospital, and the influence of above-described subpopulations on the NPV.

## Methods

### Population

**Fig. 1:**
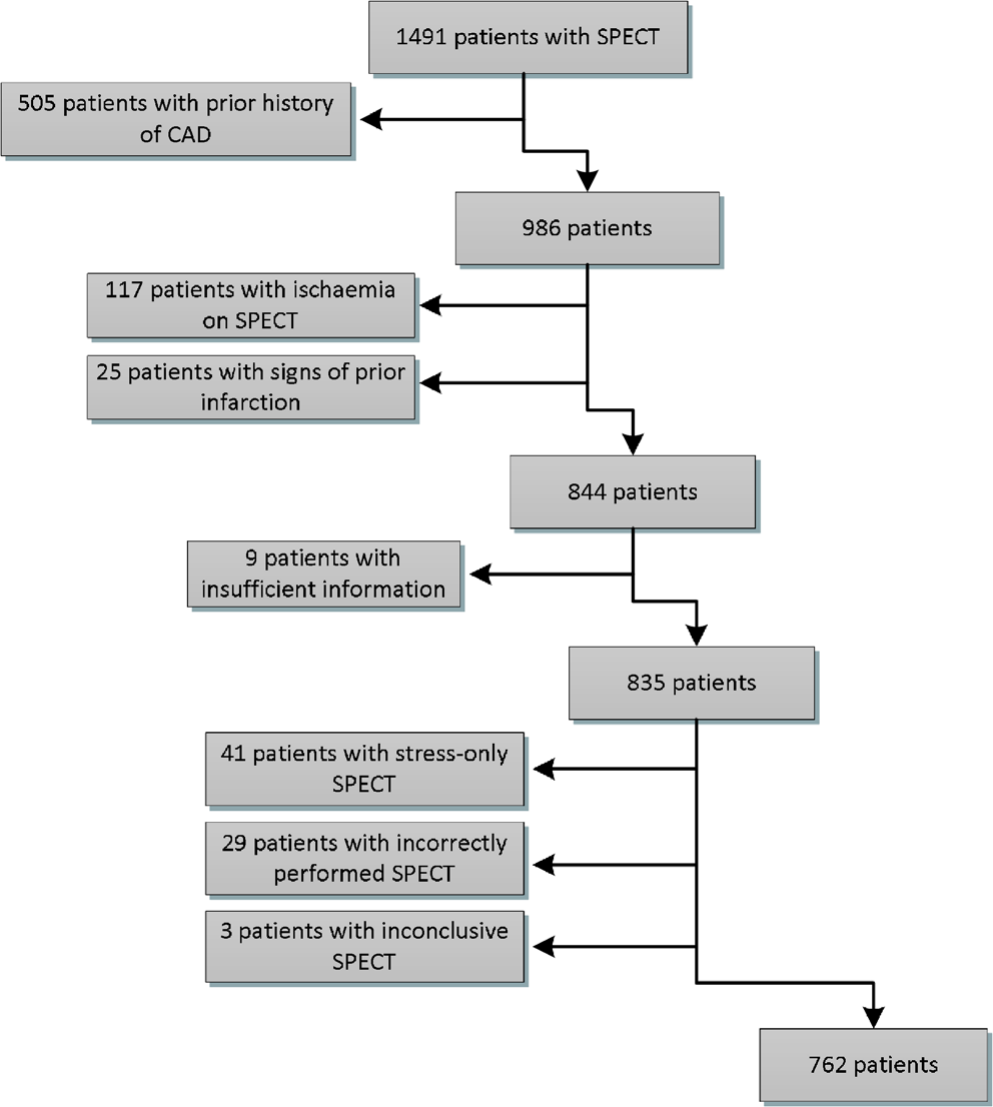
Inclusion flowchart

We identified 1491 patients who underwent SPECT between 28 April 2009 and 28 February 2011 in Deventer Hospital. Patients with a prior history of CAD were excluded, as defined by previous MI, revascularisation and/or significant stenosis on coronary angiography, as documented in the medical records. Furthermore, patients with ischaemia and with signs of prior infarction on SPECT were excluded. The remaining total number of studied patients was 762 (Fig. 1).

### Data acquisition

Data were collected from the Electronic Medical Record (EMR) by reviewing letters and reports of the treating cardiologist and other clinicians, reports and letters of clinicians in other hospitals, laboratory results, reports of SPECT by the nuclear medicine physician and the images of SPECT.

### Patient preparation and stress protocol

Patient preparation was done according to the American Society of Nuclear Cardiology protocol [19].

Adenosine (0.14 mg/kg/min) was infused intravenously for 6 min, and Tc-99m Tetrofosmine was injected after at least 3 min. Adenosine stress was combined with low level treadmill exercise, 20–70 W maximum. Contraindications for exercise were LBBB, paced rhythm and inability to exercise.

Dobutamine stress was performed in patients with severe chronic obstructive pulmonary disease. Dobutamine was administered intravenously, starting at a dose of 10 μg/kg/min. The dose was increased 10 μg/kg/min every 3 min until a heart rate of 85 % of the age-predicted maximum heart rate (220-age) was reached, with a maximum of 40 μg/kg/min. A maximum of two additional intravenous injections of atropine 0.5 mg were given.

### SPECT acquisition protocol

Data acquisition was performed using a dual head General Electric Infinia Hawkeye gamma camera (2006) with low energy high resolution collimators. Sixty projections over an angle of 180° were acquired. Acquisition times for rest and stress were 20 s and 18 s, respectively, per projection. The energy acceptance window was set at 15–20 %, 140 keV centre. An additional eight frame gated dataset was acquired with an acceptance window of 100 %. CT-based AC was used with 140 kV tube voltage and 2.5 mAs tube current. Quantitative gated SPECT software (Cedars-Sinai) was used to automatically calculate end-diastolic volume, end-systolic volume and left ventricular ejection fraction (LVEF). All non-AC images were reconstructed by filtered backward projection, using a 2D Butterworth filter with an order of 8, 0.3 cycles/pixel. AC and non-AC images were reconstructed by OSEM iterative reconstruction with 2 iterations and 10 subsets.

Patients underwent a 1-day rest-stress acquisition protocol. Rest acquisition was performed 15–30 min after intravenous injection of 250 MBq Tc-99m-tetrofosmine, stress acquisition was performed 15–30 min after intravenous injection of 750 MBq Tc-99m-tetrofosmine. A 2-day protocol was performed in 11 patients because of inadequate patient preparation and in 1 patient because of the low quality of initial stress images.

### Image interpretation

The visual interpretation of perfusion images was based on short-axis and vertical and horizontal long-axis tomograms divided into 20 segments using Myometrix (GE) software. The nuclear medicine physician reviewed all segments, in both AC and non-AC images, to review the presence of ischaemia.

### Follow-up

The follow-up time was 2 years from the time of inclusion. Patients were followed for major adverse cardiac events (MACE) and all-cause mortality. MACE was defined as fatal and non-fatal MI and revascularisation, consisting of percutaneous coronary intervention (PCI) and coronary artery bypass graft (CABG). MI was defined by typical symptoms and raised troponin-T levels. In 1 case troponin-T was not documented and MI was defined by reviewing the cardiologist letter and the CABG operation report from a tertiary clinic.

### Statistical analysis

Statistical analysis was performed with SPSS software (version 20.0.0, IBM). A *p*-value <0.05 was considered statistically significant.

To evaluate the prognosis of patients over time in the different subgroups, univariate and subsequent multivariate Cox proportional hazard regression were performed. Variables included in the univariate analysis consisted of clinical data, resting-ECG data and SPECT data. Selection of variables for entry in the multivariable Cox proportional hazard regression was based on univariate analysis (with a threshold of *p* < 0.15) and clinical judgment. The multivariate Cox regression was done stepwise according to the backwards approach, with *p* < 0.10 as threshold for removal of variables.

The rule of thumb in multivariate Cox regression models is that the events per variable (EPV) must be at least 10 to produce a reliable model [20, 21]. However, a recent study demonstrates that an EPV of 5–10 gives similar reliability to an EPV ≥10 [22]. Therefore in our study with 32 events, a prediction model may contain 3–6 variables.

The assumption of proportional hazard was tested by obtaining and reviewing the log-minus-log plots, and deemed appropriate in all categories.

To test the linearity assumption of the continuous variable age, martingale residuals were generated from the Cox regression and plotted against age. Assumption of linearity seemed appropriate.

## Results

### Study population

The mean age in the 762 included patients was 63.2 years. Of the patients 58.1 % were female and 116 (15.2 %) presented with typical chest pain. The average number of risk factors was 2.0. A total overview of clinical data, ECG data and SPECT data is shown in Table 1.

**Table 1:** Patient characteristics

*n* = 762	Number (%)
Clinical data	
Age	63.2 ± 11.9
Women	443 (58.1 %)
Clinical symptoms	
- Typical chest pain	116 (15.2 %)
- Atypical chest pain	444 (58.3 %)
- No chest pain	202 (26.5 %)
Risk factors	
- Diabetes	155 (20.3 %)
- Hypertension	389 (51.0 %)
- Family history (*n* = 750)^a^	315 (42.0 %)
- Smoking (present and former)	342 (44.9 %)
- High cholesterol	324 (42.5 %)
Total risk factors (*n* = 750)^a^	2.0 ± 1.2
Renal function (*n* = 739)^e^	
- eGFR ≥60	664 (89.9 %)
- eGFR 30–60	58 (7.6 %)
- eGFR <30	17 (2.2 %)
Cardiac history	
- Atrial fibrillation	83 (10.9 %)
- Other SVT	21 (2.8 %)
- Valvular disease	40 (5.2 %)
- Cardiomyopathy	17 (2.2 %)
- Heart failure, systolic	17 (2.2 %)
- Pacemaker placement	10 (1.3 %)
- Valve operation	8 (1.0 %)
- Heart failure, diastolic	7 (0.9 %)
- High grade AV block	3 (0.4 %)
- Ventricular tachycardia	2 (0.3 %)
- ICD	1 (0.1 %)
Resting ECG	
- Signs of prior MI	18 (2.4 %)
- Signs of ischaemia	122 (16.0 %)
- Atrial fibrillation	67 (8.8 %)
- Left bundle branch block	19 (2.5 %)
SPECT data	
Type of stress	
- Adenosine + exercise	611 (80.2 %)
- Adenosine only	125 (16.4 %)
- Dobutamine only	14 (1.8 %)
- Dobutamine + atropine	10 (1.3 %)
- Exercise + atropine	2 (0.3 %)
SPECT with AC	753 (98.8 %)
Positive exercise ECG at SPECT (*n* = 760)^c^	150 (19.7 %)
Triple vessel disease not excluded^e^	39 (5.1 %)
Summed difference score (SDS) >2	127 (16.5 %)
Functional data	
- LVEF <45 (*n* = 758)^d^	41 (5.4 %)
- EDV >120	113 (14.8 %)
- ESV >60	89 (11.7 %)

*eGFR* estimated glomerular filtration rate, *Other SVT* AV nodal re-entry tachycardia/atrial flutter, *ICD implantable cardioverter defibrillator*, *AC attenuation correction*, *LVEF* left ventricular ejection fraction, *EDV* end-diastolic volume, *ESV* end-systolic volume, *Triple vessel disease not excluded* due to ventricular wall motion abnormalities or abnormal functional data

^a^In 12 cases family history was not obtained

^b^eGFR was not obtained in 23 patients

^c^In two patients stress ECG was not obtained due to technical difficulties

^d^In four cases LVEF was unreliable, due to AF or GI activity

^e^Triple vessel disease could not be excluded due to wall motion abnormalities

### Outcome

Of the 762 studied patients, 32 (4.2 %) had MACE within follow-up: 1 patient (0.1 %) died of MI, 8 (1.0 %) patients had non-fatal MI, in 12 (1.6 %) patients CABG was performed and 21 (2.8 %) received PCI. The all-cause mortality in our study population was 15 (2.0 %). The NPV of SPECT for the occurrence of MACE within 2 years was 95.8 % and the NPV for MI only was 98.8 %. An overview of MI, MACE and the NPV of SPECT in the various subgroups is given in Table 2.

**Table 2:** Outcome and negative predictive value

	MI (%)	MACE (%)	NPV for MACE
Overall	9 (1.2 %)	32 (4.2 %)	95.8 %
Women	1 (0.2 %)	8 (1.8 %)	98.2 %
AF	2 (1.9 %)	2 (1.8 %)	98.2 %
DM	2 (1.3 %)	9 (5.8 %)	94.2 %
LBBB	1 (1.9 %)	4 (7.7 %)	92.3 %
Renal impairment	2 (2.7 %)	4 (5.3 %)	94.7 %
Severe renal impairment	1 (5.9 %)	2 (11.8 %)	88.2 %

*AF* atrial fibrillation (prior history/on ECG at presentation), *DM* diabetes mellitus, *LBBB* left bundle branch block, *renal impairment* eGFR<60, *severe renal impairment* eGFR<30

### Prediction model for the occurrence of MACE

Univariate and subsequent stepwise multivariate Cox proportional hazard regression was performed and the results are presented in Table 3. The variables with a *p*-value <0.15 on univariate analysis and the clinically important variables AF, DM, renal impairment and LBBB were included in the multivariate Cox regression. The only significant independent predictors of MACE were male gender, positive stress ECG on SPECT and low LVEF. AF and hypertension tended to be independent predictors, but were not significant.

**Table 3:** Univariate and subsequent multivariate Cox regression of clinical data, ECG findings and SPECT data on MACE

Variable	HR	95 % CI	*P*-value
Univariate analysis			
Male gender	4.266	1.916–9.496	<0.001*
Age	1.021	0.990–1.052	0.189
Typical chest pain	1.885	0.847–4.196	0.120*
DM	1.523	0.705–3.292	0.285
Hypertension	1.856	0.895–3.850	0.097*
Family history	1.222	0.610–2.447	0.572
Smoking	1.088	0.544–2.179	0.811
High cholesterol	1.189	0.594–2.380	0.625
Renal impairment	1.272	0.446–3.625	0.653
Severe renal impairment	2.917	0.697–12.207	0.143*
AF	0.403	0.096–1.687	0.213
ECG: prior MI	1.330	0.182–9.744	0.779
ECG: ischaemia	0.535	0.163–1.755	0.302
ECG: LBBB	2.021	0.709–5.761	0.188
Adenosine only	0.799	0.327–1.955	0.623
Stress ECG at SPECT	2.257	1.082–4.711	0.030*
LVEF < 45 %	4.369	1.798–10.615	0.001*
ESV > 60	2.165	0.937–5.006	0.071*
EDV > 120	3.110	1.500–6.451	0.002*
Triple vessel disease not excluded	3.638	1.401–9.448	0.008*
Multivariate analysis			
Male gender	4.710	2.054–10.801	<0.001
Hypertension	1.960	0.936–4.105	0.074
Stress ECG at SPECT	3.079	1.460–6.495	0.003
LVEF < 45 %	3.967	1.584–9.934	0.003
AF	0.303	0.071–1.294	0.107

*LVEF* left ventricular ejection fraction, *EDV* end-diastolic volume, *ESV* end-systolic volume, *DM* diabetes mellitus, *AF* atrial fibrillation (prior history/on ECG at presentation), *MI* myocardial infarction, *LBBB* left bundle branch block, *Renal impairment* eGFR<60, *severe renal impairment* eGFR<30

*: variables with *p* < 0.15 on univariate analysis

## Discussion

### Outcome

Our results show that the prognosis of combined stress SPECT with AC without perfusion deficits is very good in patients without a prior history of CAD, with an NPV for the occurrence of MACE within 2 years of 95.8 % and an NPV for MI of 98.8 %.

Statistical analysis showed that besides positive stress ECG at SPECT and low LVEF, male gender was the only significant independent predictor for the occurrence of MACE. Thus, the prognosis of a negative SPECT result is not altered by AF, diabetes, LBBB and renal impairment.

### Overall population

The prognosis after normal SPECT in patients without prior history of CAD has not been previously researched in terms of MACE-free survival. Our study therefore provides key information for the use of SPECT in a medium-sized clinic in the Netherlands. Only two studies are published evaluating the prognosis of a normal SPECT in patients without prior history of CAD in terms of MI rate. These studies report an annual MI rate of 0.6 % following a normal SPECT result [6, 23], which is in accordance with the MI rate of 1.2 % within 2 years found in our study.

### Subgroups

The greater prognostic value of SPECT in women compared with men, as seen in our study, has not previously been reported. Prior studies even show that women have a slightly higher event rate than men after normal SPECT [7]. These studies are predominantly performed in large clinics outside the Netherlands. Our results may be more representative for clinics in the Netherlands. Research in the Leiden University Medical Centre showed a worse prognosis of MACE in 5.9 % of patients [24]. This study, however, was performed in a higher risk population and with a broader definition of MACE. The worse prognosis in men is an important finding for the daily practice in our hospital and in comparable clinics. Clinicians should be more careful in interpreting normal SPECT in men.

Unlike our results, prior studies show a higher event rate in AF patients after normal SPECT than in non-AF patients [15]. These studies defined AF only by findings on the baseline resting ECG. Both paroxysmal and persistent AF are known to have increased risk of developing MACE [25, 26]. Therefore, we also included patients with a prior history of AF to analyse the NPV of SPECT in AF patients. Our study is the first to show that the prognosis of normal SPECT in AF patients (on baseline resting ECG and prior history) is comparable with that of non-AF patients.

Patients with renal impairment (eGFR<60) are reported to have a worse prognosis after normal SPECT than patients with normal renal function [11–13]. Our study, however, did not show any difference in MACE-free survival between these patients. Although patients with severe renal impairment (eGFR<30) seemed to have a worse prognosis, with MACE in 11.8 % of patients, no statistically significant difference was proven.

The similar prognosis of diabetics and non-diabetics after normal SPECT is in accordance with prior studies [16]. However, the event rate after normal SPECT, although initially similar, is reported to increase after 2 years of follow-up and thus a shorter warranty period of SPECT may apply in diabetic patients [16]. One must be cautious about using our data to predict the long-term prognosis of a normal SPECT in diabetic patients.

Patients with LBBB are known to have an abnormal conduction pattern, associated with abnormal perfusion images. When this is taken into account, patients with LBBB are reported to have a similar prognosis after normal SPECT to patients without LBBB [18]. Although the prognosis seemed worse, with MACE in 7.7 % of LBBB patients, compared with 2.6 % in non-LBBB patients, no difference in MACE-free survival was found. However, the small cohort of 52 LBBB patients limits the extrapolation of results and therefore we advise clinicians to be cautious when reviewing normal SPECT in LBBB patients.

### Limitations

Recently, software has been developed to automatically quantify perfusion in SPECT. The automated quantitative scores are reported to have greater prognostic value [27] and reproducibility than visual assessment [28]. In our study, perfusion images of SPECT were visually assessed, because local experience with the visual approach was greater than with automated quantitative software. Although SPECT is known to have little intra- and inter-observer variability in experienced observers [3, 29, 30], the reliance upon the expertise of the observer limits the extrapolation of our results to other centres.

The definition of MI was also subject to inter-observer variability, because the cardiologist’s interpretation and clinical judgement played an important role in determining whether the patient presented with classical signs of MI.

Our population of 762 patients was sufficient to investigate the NPV of a negative SPECT in the overall population, despite the limited number of events. However, the number of patients in the various subgroups was often small. To prove statistically significant differences in MACE-free survival in subgroups larger study populations are needed, especially in patients with LBBB and patients with severe renal impairment. The limited number of events also compromised the reliability of the prediction model generated by the Cox proportional hazard regression.

## Conclusion

Our study shows that the negative predictive value of combined stress SPECT with AC on the occurrence of MACE within 2 years in patients without prior CAD in a medium-sized clinic in the Netherlands is very good, even in patients with AF, diabetes and moderate renal impairment. However, caution is advised when reviewing SPECT in men, patients with LBBB and patients with severe renal impairment. Statistical analysis showed significantly worse MACE-free survival in men. MACE-free survival also seemed worse in patients with LBBB and patients with severe renal impairment, but this was not significant. Further prospective research is needed in these patients.